# Flexible Surface Acoustic Wave Sensor Based on an SXFA Sensitive Film for DMMP Detection

**DOI:** 10.3390/polym18212651

**Published:** 2026-10-26

**Authors:** Cancan Yan, Yanze Wang, Junchao Yang, Jie Yang, Lin Zhang, Yong Pan, Genwei Zhang

**Affiliations:** State Key Laboratory of Chemistry for NBC Hazards Protection, Beijing 102205, China; ccy805905145@163.com (C.Y.); 13651334656@163.com (Y.W.); yangjunchao1990@163.com (J.Y.); lin_44627079@yeah.net (J.Y.); zhanglin_zju@aliyun.com (L.Z.)

**Keywords:** surface acoustic wave, flexible sensor, SXFA, hexafluoroisopropyl, DMMP, hydrogen-bond acidic polymer, gas sensor

## Abstract

Rapid and reliable detection of organophosphorus nerve agents is essential for public safety and individual protection. In this work, a flexible surface acoustic wave (SAW) gas sensor based on a hexafluoroisopropyl (HFIP)-functionalized polysiloxane (SXFA) sensitive film was developed for the detection of dimethyl methylphosphonate (DMMP), a widely used simulant of sarin (GB). SXFA was synthesized through a two-step route and systematically characterized by Fourier-transform infrared spectroscopy (FTIR), ^1^H/^19^F/^13^C nuclear magnetic resonance (NMR), gel permeation chromatography (GPC), differential scanning calorimetry (DSC), thermogravimetric analysis (TGA) and scanning transmission electron microscopy coupled with energy-dispersive X-ray spectroscopy (STEM/EDX). The results confirm the successful incorporation of HFIP groups and reveal a low glass-transition temperature (*Tg* = −23.5 °C, ΔCp = 0.295 J·g^−1^·K^−1^), a weight-average molecular weight of ~3200 g·mol^−1^, a thermal-stability limit of ~200 °C, and a characteristic HFIP side-chain cleavage at 343.4 °C with a final residue of 18.37%. The flexible device consisted of a polyimide (PI) substrate, a magnetron-sputtered AlN piezoelectric layer and silver interdigital transducers (IDTs) and operated at a center frequency of ~200 MHz; a rigid quartz-based delay line was used as a reference. The dependence of the insertion loss (IL) on film thickness was described by a third-order polynomial (*R*^2^ = 0.960), from which an optimal thickness window of 150–450 nm was identified, and 450 nm gave the best gas-sensing performance (response 23.5 mV at 3 mg·m^−3^ DMMP, IL −16.0 dB; mean ± SD, *n* = 3 independently prepared devices per thickness). The optimized sensor exhibited a linear response to DMMP from 0.4 to 6 mg·m^−3^ (y = 30.88x + 8.988, *R*^2^ = 0.9985) at 28.1 °C, fast response and recovery (~75 s each), excellent reproducibility (relative standard deviation, RSD = 0.50%, *n* = 7), pronounced selectivity towards DMMP and DFP over common interferents, and a 60-day response retention of 96.0%. The sensing mechanism is attributed to hydrogen-bonding adsorption between the acidic HFIP groups and the basic P=O moiety of DMMP, combined with mass-loading and film-viscoelastic effects. These results demonstrate that SXFA-functionalized flexible SAW devices are promising candidates for wearable and low-power detection of organophosphorus nerve agents.

## 1. Introduction

Organophosphorus (OP) compounds constitute the most important class of chemical warfare agents (CWAs) and remain a serious threat to public security, military operations and civilian safety [1,2]. Sarin (GB), soman (GD) and VX are typical G- and V-series nerve agents whose acute toxicity originates from the irreversible inhibition of acetylcholinesterase, which leads to cholinergic crisis and, at sufficient doses, to death within minutes [1,3]. The Tokyo subway attack in 1995 and several subsequent incidents demonstrated that such agents can be dispersed in confined public spaces with devastating consequences, and they remain a persistent concern in demilitarization, counter-terrorism and environmental monitoring scenarios [2,3]. Because the direct handling of nerve agents is restricted to authorized laboratories, dimethyl methylphosphonate (DMMP, CH_3_P(=O)(OCH_3_)_2_) is widely adopted as a safe structural simulant of GB. DMMP reproduces the key physicochemical feature of nerve agents, a strongly basic phosphoryl (P=O) oxygen together with a methylphosphonate backbone, while its lower toxicity permits routine laboratory experimentation. Consequently, DMMP is the standard probe molecule for evaluating the performance of nerve-agent gas sensors [4,5,6]. Conventional analytical techniques, including ion-mobility spectrometry, gas chromatography–mass spectrometry, flame photometry and colorimetric or fluorescence methods, provide high sensitivity and selectivity, but they generally rely on bulky, expensive instrumentation, require skilled operators and are difficult to deploy for continuous, on-body monitoring [7,8]. For point-of-care and wearable applications, a compact, low-cost, passive and fast-responding gas-sensing platform is therefore highly desirable.

Surface acoustic wave (SAW) devices, first demonstrated by White and Voltmer in 1965 [9], have since become one of the most versatile platforms for chemical vapor sensing [10,11,12]. In a SAW gas sensor, an interdigital transducer (IDT) patterned on a piezoelectric medium converts an electrical signal into an acoustic wave that propagates along the device surface and is reconverted into an electrical signal by a second IDT. A sensitive film deposited on the propagation path reversibly sorbs target molecules, and the resulting perturbation of the wave velocity, attenuation and resonance frequency provides a measurable readout [10,11]. Compared with other transducing schemes, SAW sensors offer high mass sensitivity, a small footprint, low power consumption, room-temperature operation and compatibility with wireless/passive interrogation [12,13,14,15]. Sensitive-film materials play a decisive role in determining sensitivity and selectivity [16]. Among them, polymers are particularly attractive because they are non-volatile, are readily deposited by spin- or drop-coating, and provide a viscoelastic matrix in which analyte molecules can rapidly diffuse and partition [16,17].

The most successful class of polymer coatings for OP detection is the hydrogen-bond acidic (HBA) family, whose selectivity can be rationalized quantitatively through the linear solvation energy relationship (LSER) [18,19]:

(1) log *K* = *rR*_2_ + *sπ*_2_^*H*^ + *aα*_2_^*H*^ + *bβ*_2_^*H*^ + *l* log *L*^16^

Here, *K* is the gas–polymer partition coefficient; *c*, *r*, *s*, *a*, *b* and *l* are fitted constants; and *R*_2_, *π*_2_^*H*^, *α*_2_^*H*^, *β*_2_^*H*^ and *L*^16^ denote the excess molar refraction, dipolarity/polarisability, hydrogen-bond acidity, hydrogen-bond basicity and hexadecane–air partition coefficient of the analyte, respectively [18]. For hydrogen-bond basic analytes such as GB, an ideal coating should possess the largest possible b together with the smallest possible a and polarity s. SXFA—poly(4-hydroxy-4,4-bis(trifluoromethyl)butyl-1-enyl)siloxane—is the most widely adopted member of the HBA polysiloxane family [20,21]. Every repeat unit bears a hexafluoroisopropyl (HFIP) pendant, and the two flanking –CF_3_ substituents withdraw electron density so strongly that the hydroxyl proton becomes markedly acidic. Consequently, the polymer readily forms strong hydrogen bonds with the basic phosphoryl oxygen that is common to organophosphorus vapors. The LSER selectivity ratios reported for this coating (b/a = 6.07, b/s = 7.08, s/a = 1.17) place it firmly within the optimum window for HBA sensing layers [4,18]. Equally important, its glass-transition temperature lies far below ambient, so that the film stays rubbery and viscoelastic during operation, a prerequisite for rapid analyte uptake and complete release.

These attributes have prompted extensive study of SXFA in SAW-based OP detection. A 200 MHz shear-horizontal delay line fabricated on Y-cut quartz and coated with SXFA detected GB below 0.1 mg·m^−3^ [4], while a 433 MHz wireless passive SAW platform built on YZ-LiNbO_3_ and functionalized with the same polymer responded to DMMP down to 0.48 mg·m^−3^, with a sensitivity of 4.63°/(mg·m^−3^) [22]. In both cases, however, the device rested on a rigid and brittle substrate, which restricts conformal attachment to curved or moving surfaces and therefore limits its use in wearable systems.

Flexible acoustic wave devices have recently emerged as a route to wearable and conformal sensing [23,24,25,26]. By replacing the rigid substrate with a thin polymer foil—most commonly polyimide (PI), polyethylene naphthalate (PEN) or polyethylene terephthalate (PET)—and depositing a piezoelectric thin film, such as ZnO, AlN or PZT, on top, devices that can be bent, wrapped and attached to curved surfaces have been demonstrated [23,25,27,28,29,30,31,32]. Such flexible SAW platforms have been reported for temperature, humidity, strain and magnetic sensing [26,29,33,34], and the recent review literature has highlighted chemical gas sensing as the most promising, yet least explored, application of flexible SAW technology [23]. Two key issues govern the performance of a flexible SAW chemical sensor. The first is the acoustic loss introduced by the polymer-sensitive film: although a thicker film offers more sorption sites, it also imposes a heavier mass and viscoelastic load that increases the insertion loss and can eventually suppress the resonant response [35]. The second is the intrinsic conflict between the soft, mechanically compliant substrate and the requirement of stable acoustic propagation. Both effects must be quantified before flexible SAW sensors can be engineered reliably.

In this work, we address these challenges by integrating the benchmark HBA polymer SXFA with a flexible SAW delay line fabricated on a polyimide substrate. SXFA was comprehensively characterized by FTIR, NMR, GPC, DSC, TGA and STEM/EDX, a level of analysis unprecedented for SXFA in a flexible SAW context. The SXFA-coated device was systematically evaluated for insertion loss and DMMP sensing performance (response dynamics, linearity, reproducibility, long-term stability and selectivity), and the sensing mechanism was interpreted in terms of hydrogen-bonding adsorption, mass loading and film viscoelasticity.

## 2. Materials and Methods

### 2.1. Materials and Device Fabrication

Allylmethyldichlorosilane (95%) and hexafluoroacetone trihydrate (HFA·3H_2_O, 95%) were purchased from Macklin Biochemical Co., Ltd.(Shanghai, China). Phenyltrimethylammonium hydroxide (20–25% in methanol), anhydrous diethyl ether (99.9%), toluene (AR), magnesium sulfate (AR), sulfuric acid (AR) and ethanol (≥99.7%) were obtained from Sinopharm Chemical Reagent Co., Ltd. (Beijing, China) and used as received. DMMP (AR) was supplied by Beijing Chemical Plant (Beijing, China), and deionized water (18.2 MΩ·cm) was used throughout. A polyimide (PI) foil (25 μm) together with AlN and Ag sputtering targets (both 99.99%) were purchased from Zhongnuo Advanced Material Technology Co., Ltd. (Beijing, China)served for device fabrication.

SXFA was prepared by the two-step synthesis reported in our previous work [4], which should be consulted for the full procedure, for the characterization of the intermediates and for the reaction mechanism. In brief, allylmethyldichlorosilane was hydrolyzed and condensed in anhydrous diethyl ether to give a methylvinylsiloxane precursor, which was aged, ring-opened with phenyltrimethylammonium hydroxide and equilibrated to a high-molar-mass methylvinylpolysiloxane. Hydrolysis of allylmethyldichlorosilane yields cyclic siloxane oligomers; therefore, the strong base phenyltrimethylammonium hydroxide is required to perform ring opening and subsequent equilibration to build high-molar-mass linear methylvinylpolysiloxane. It should be noted that the reactive side group of the synthesized methylvinylpolysiloxane is vinyl (–CH=CH_2_, vinylic group), rather than an allylic group, despite the allyl-containing starting silane monomer. Hexafluoroacetone, liberated from its trihydrate over concentrated H_2_SO_4_, was then added across the vinyl groups in a sealed ampoule at 380 K for 48 h to give the target polymer, poly(4-hydroxy-4,4-bis(trifluoromethyl)butyl-1-enyl)siloxane.

The flexible device was configured as a delay line. An AlN piezoelectric thin film was deposited onto the PI foil by radio-frequency magnetron sputtering from a stoichiometric AlN target, and a Ag layer was subsequently deposited by direct-current magnetron sputtering and patterned into interdigital transducers (IDTs) by conventional ultraviolet photolithography and lift-off. Photoresist and developer for photolithography were obtained from Suzhou Ruihong Electronic Chemicals Co., Ltd. (Suzhou, China).A pair of unidirectional IDTs with electrode widths of ~4 μm and ~2 μm was employed to obtain a low-loss, single-mode response. The fundamental resonance frequency (~200 MHz) is mainly determined by the IDT periodicity, while the input–output spacing defines the acoustic propagation path length. The AlN piezoelectric layer thickness is 1.2 μm, with c-axis preferred orientation; Ag IDT electrode thickness is 200 nm. Other device parameters are: IDT periodicity 10 μm, finger-pair count 80, acoustic propagation length 1200 μm, radio-frequency magnetron sputtering power 250 W, N_2_/Ar gas ratio 1:2, and working pressure 0.4 Pa for AlN deposition. A 50 nm SiO_2_ layer was finally sputtered over the transducers to protect them during polymer coating. The SiO_2_ sputtering target (99.99%) was purchased from Zhongnuo Advanced Material Technology Co., Ltd. (Beijing, China).For reference, a rigid delay line with identical IDT geometry and nominal operating frequency was fabricated on Y-cut quartz(Jingshi Quartz Technology Co., Ltd. (Lianyungang, China) and characterized in parallel; these data serve only to benchmark the electrical behavior of the flexible device.

### 2.2. Film Deposition and Characterization

SXFA films of controlled thickness were deposited on the delay path by spin coating (for the sensing devices) or drop-casting (for the material specimens) from dilute toluene solutions. Spin coating was performed using an SCS G3P-8 coating system(Specialty Coating Systems, Inc., Indianapolis, IN, USA); spin coating parameters were: two step procedure, first step 500 rpm for 10 s, and second step 2500 rpm for 30 s from dilute toluene SXFA solutions. The film thickness was determined by stylus profilometry and cross-checked against the calibrated film-thickness–insertion-loss relationship; nominal thicknesses of 150, 300, 450, 600, 750, 900, 1050, 1200 and 1350 nm were investigated.

FTIR spectra were recorded in transmission mode on an FTS-185 spectrometer (Bio-Rad Laboratories, Inc., Hercules, CA, USA) over 4000–400 cm^−1^. Solution NMR spectra were acquired on a Bruker AVANCE III400 MHz spectrometer (Bruker Corporation, Billerica, MA, USA): ^1^H at 400 MHz, ^19^F at 376 MHz and ^13^C at 100 MHz, with CDCl_3_ as the solvent and internal reference. GPC was performed on a Shimadzu LC-20AT system with a differential refractive-index detector and polystyrene calibration. DSC was carried out on a NETZSCH DSC 300 Classic calorimeter(Shimadzu Corporation, Kyoto, Japan) with 4.0 mg samples in perforated concave aluminum crucibles under high-purity N_2_ (purge/protective flows of 250 mL·min^−1^), using a three-segment program (−85 → 80 → −85 → 80 °C at 10 K·min^−1^). TGA was performed on a NETZSCH STA 449F3 instrument(NETZSCH-Gerätebau GmbH, Selb, Germany) with 1.9294 mg of sample in an Al_2_O_3_ crucible under N_2_ from 27 to 800 °C at 10.0 K·min^−1^. Talos F200X STEM(Thermo Fisher Scientific, Eindhoven, The Netherlands), high-angle annular dark-field (HAADF) imaging, selected-area electron diffraction (SAED) and EDX mapping were performed on SXFA nanoparticle specimens dispersed on a carbon-coated copper grid.

Two-port S-parameters (S_21_) of the bare and SXFA-coated devices were recorded with a Keysight E5061B vector network analyzer(Keysight Technologies, Santa Rosa, CA, USA) over 195–205 MHz, and the steady-state insertion loss at the main resonance was extracted and plotted against the nominal film thickness.

Bending tolerance of the completed flexible device was evaluated on a dedicated three-point bending stage. The as-fabricated device (PI substrate carrying the sputtered AlN layer, the Ag IDTs and the SXFA-coated delay path) was clamped in the fixture and deflected to bending angles of 0°, 10°, 20°, 30° and 35°, with the bending axis aligned both parallel and perpendicular to the SAW propagation direction; the corresponding bending radii follow from the fixture geometry. Cyclic bending was performed for 1000 cycles at the maximum deflection. S_21_ transmission spectra, the phase-detector baseline and the DMMP response were recorded in the flat state, under deflection and after release.

### 2.3. Gas-Sensing Measurements

DMMP vapor was generated with a calibrated dynamic gas generator and diluted with dry air to the required concentration. All sensing measurements were conducted under stable conditions at 27.8–28.1 °C with constant relative humidity. DMMP vapor was delivered into the sealed gas chamber via controlled dilution gas streams; the flow rate and concentration ratio were regulated according to the pre-established calibration curve. The sensor was mounted in a sealed gas chamber and interrogated by a phase-detection circuit: a 200 MHz reference signal and the signal transmitted through the delay line were combined in a phase detector, whose output voltage is proportional to the phase difference and therefore to the accumulated acoustic perturbation. The signal was logged through a data-acquisition module. For each measurement cycle, DMMP-laden air was introduced for a fixed exposure period, and dry air was then supplied for recovery, so that the response was recorded as the change in the phase-detector output voltage (mV) relative to the dry-air baseline. Selectivity was evaluated by exposing the sensor to DMMP, DFP, NH_3_, dimethylacetamide (DMAC), ethanol, acetone and methanol at a common concentration of 5 mg·m^−3^. Long-term stability was assessed by measuring the response to 5 mg·m^−3^ DMMP at 28.1 °C at intervals over 60 days of ambient storage. All thickness-dependent response measurements in Section 3.3 were performed at a fixed DMMP concentration of 0.8 mg·m^−3^. For each film thickness, three independent SAW devices with identically deposited SXFA films were fabricated and measured to obtain the mean response and standard deviation for error-bar plotting.

## 3. Results and Discussion

The results are presented in three stages. Section 3.1 and Section 3.2 establish the chemical identity, the thermal behavior and the nanoscale morphology of the synthesized SXFA, that is, the material properties that ultimately govern the sensing response. Section 3.3 then turns to the flexible delay line itself, describing its transmission characteristics, quantifying how the polymer coating and the film thickness perturb acoustic propagation, and identifying the optimum coating thickness. Section 3.4, Section 3.5, Section 3.6 and Section 3.7 evaluate the DMMP-sensing performance of the optimized device, interpret that performance in terms of the underlying sorption and transduction mechanisms, and finally benchmark it against previously reported organophosphorus SAW sensors.

### 3.1. Chemical Structure of SXFA (FTIR and NMR)

Figure 1a shows the FTIR spectrum of the synthesized SXFA, and the assignment of the principal absorption bands is summarized in Table 1. The strong, broad band centered at 1010.7 cm^−1^ with a shoulder at 1148.9 cm^−1^ arises from the Si–O–Si stretching vibration of the polysiloxane backbone and is the most intense feature of the spectrum, as expected for a polysiloxane. The band at 1267.9 cm^−1^ is assigned to the overlapping C–F stretching and Si–CH_3_ symmetric deformation vibrations, while the two bands at 1210.0 and 1187.7 cm^−1^ together with the band at 538.7 cm^−1^ are characteristic of the –CF_3_ groups of the HFIP moiety. The absorption at 918.8 cm^−1^ (Si–OH) and 777.1 cm^−1^ (Si–C) confirms the siloxane skeleton, and the bands at 1604.3, 733.1 and 701.4 cm^−1^ are attributed to the aromatic C=C stretching and C–H out-of-plane/ring-deformation modes. Notably, the presence of the –CF_3_ bands between 1210 and 1120 cm^−1^, together with the persistence of a Si–OH absorption, indicates that the addition of HFA to the vinyl groups of the precursor polysiloxane has taken place, generating the HFIP functionality; this is consistent with the spectroscopic fingerprint reported previously for SXFA [4,20]. Note that the intensity of broad O H stretching absorption near 3400 cm^−1^ differs compared with our earlier report. This difference originates from different sample preparation conditions: the earlier measurement was performed with a KBr pellet powder sample, while the present FTIR was measured on a spin cast thin film specimen. Although the broad 3400 cm^−1^ O H stretching band is attenuated in thin film mode, the diagnostic Si OH band at 918.8 cm^−1^ is still clearly observable, confirming that hydroxyl groups are retained within the SXFA material. All peak assignments for aromatic carbons and –O–CH_2_– groups below are justified with the repeat-unit chemical structure of SXFA shown in Figure S1.

The molecular structure was further confirmed by multinuclear NMR (Figure 1b). In the ^1^H spectrum, the singlet at δ = 0.00 ppm is assigned to the Si–CH_3_ protons of the backbone, the resonance at δ = 1.84 ppm is assigned to the aliphatic –CH_3_ groups, the signal at δ = 3.20 ppm is assigned to the –O–CH_2_– methylene protons, and the peaks at δ = 5.40 and 6.48 ppm are assigned to vinylic/aromatic =C–H protons. The ^19^F spectrum displays two well-resolved resonances at δ = −76.72 and −79.02 ppm, unambiguously assigned to the –C(CF_3_)_2_–OH and the –CF_3_ fluorine nuclei of the HFIP group, respectively. In the ^13^C spectrum, the signals at δ = 134.84 and 122.02 ppm correspond to the aromatic/vinylic C=C carbons. These resonances at δ 119–135 ppm arise from residual PTMA end-capping units of the precursor, rather than the main chain of SXFA. The resonance at δ = 76.60 ppm is assigned to the C–O/C–F carbons of the HFIP unit, and the peaks at δ = 35.21 and 29.51 ppm are assigned to the –CH_2_– methylene carbons; the peak at δ = 76.60 ppm is clearly distinguished from the residual CDCl_3_ solvent peak at δ = 77.16 ppm. We note that fluorine resonances and Si–OH absorption alone cannot confirm covalent HFIP attachment. The covalent grafting conclusion is supported by combined evidence from FTIR, ^1^H, ^13^C and ^19^F NMR spectra. The signal at δ ≈ 77 ppm is the CDCl_3_ reference. The simultaneous observation of the ^19^F HFIP signature and the ^13^C C–O/C–F resonance provides direct evidence that the HFIP functional groups, the sites responsible for hydrogen-bond acidity, are covalently attached to the polysiloxane backbone. We note that these aromatic related signals at δ 119–135 ppm originate from residual PTMA end-capping units from polymer synthesis and are not assigned to residual phenyltrimethylammonium hydroxide impurity. Phenyl-containing cations from this base would produce distinct diagnostic NMR shifts, which are absent in our spectra, so significant residual phenyltrimethylammonium hydroxide impurity can be ruled out for the measured SXFA sample.

**Figure 1:**
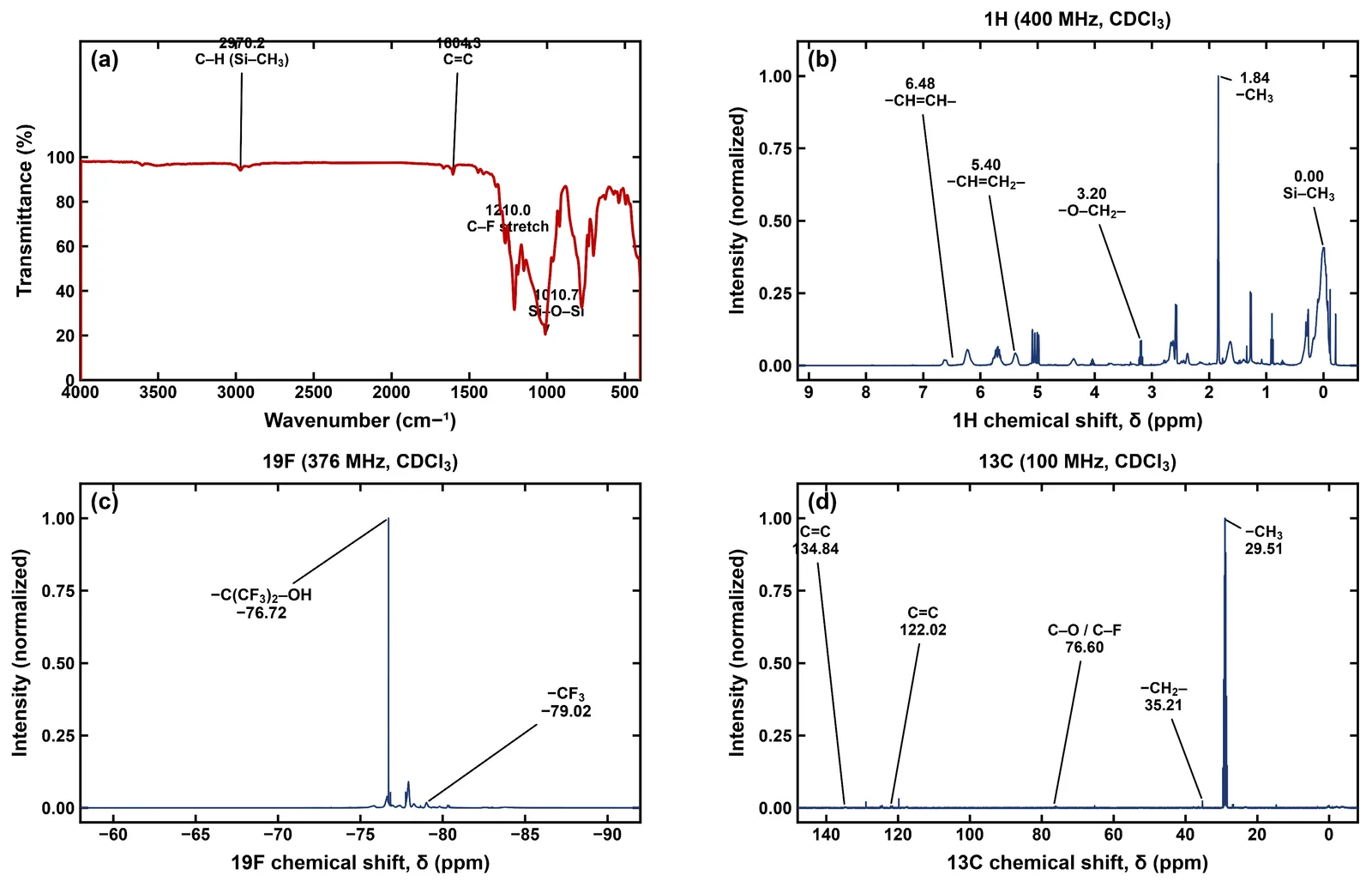
Spectroscopic characterization of the synthesized SXFA. (**a**) FTIR transmission spectrum (4000–400 cm^−1^); the band assignments are listed in Table 1. (**b**–**d**) ^1^H (400 MHz), ^19^F (376 MHz) and ^13^C (100 MHz) NMR spectra acquired in CDCl_3_, with the chemical shifts of the individual resonances indicated. The two ^19^F resonances at δ = −76.72 and −79.02 ppm and the ^13^C signal at δ = 76.60 ppm are diagnostic of the hexafluoroisopropyl (HFIP) unit. Together with FTIR and ^1^H/^13^C NMR data, these peaks collectively demonstrate that the HFIP groups are covalently bound to the polysiloxane backbone.

### 3.2. Molecular Weight, Thermal Properties and Morphology

The GPC trace of SXFA exhibited a single, narrow, monomodal peak at a retention time of 9.051 min (peak width 8.067–9.542 min), with an area fraction of 100% and a polydispersity consistent with a well-defined oligomer. Calibration with polystyrene standards placed the weight-average molecular weight at approximately 3200 g·mol^−1^, corresponding to a degree of polymerization of about 9. This relatively low molecular weight is typical of SXFA sensing grades: it preserves solution processability and enables homogeneous, pinhole-free thin films while keeping the chain-end (Si–OH) content and hence the glass-transition temperature at levels favorable for rapid analyte diffusion [4].

The thermal behavior of SXFA is presented in Figure 2 and Table 2. The DSC curves (Figure 2a) show the characteristic first-heating, cooling and second-heating segments. The first heating trace is dominated by a broad endothermic relaxation below −55 °C associated with the release of residual internal stress and erasure of the thermal history. In the cooling segment, the heat flow remains negative throughout, and no exothermic crystallization peak is observed, indicating that SXFA does not crystallize and stays amorphous on cooling, behavior consistent with the low crystallinity of polysiloxanes. The second-heating curve, which represents the intrinsic thermal behavior, exhibits a clear step-like change in heat capacity: the transition begins at about −27 °C, passes through a midpoint at −23.5 °C and ends at about −19 °C, from which a glass-transition temperature *T*g(mid) = −23.5 °C and a heat-capacity increment Δ*C*p = 0.295 J·g^−1^·K^−1^ were determined. This low *T*g means that, at ambient temperature, the SXFA film is well above its glass transition and therefore remains rubbery and viscoelastic, which is a prerequisite for fast sorption/desorption kinetics and for the swelling-based response mechanism exploited in SAW vapor sensing [17].

Five successive weight-loss events are resolved in the TGA/DTG curves (Figure 2b). Between 27 and 200 °C, the mass falls slowly and almost linearly (−11.3%) without any resolvable DTG maximum; physically desorbed water and residual solvent account for this stage. The following interval (200–240 °C, −5.93%) produces only a faint shoulder on the DTG trace and is attributed to the chain-end groups and low-molar-mass oligomers already detected by GPC. The corresponding GPC chromatogram is provided in Figure S2 of Supporting Information for visual reference. The first substantial decomposition then sets in between 240 and 350 °C, removing a further 30.97% of the mass; its DTG maximum at 343.4 °C (−3.47%·min^−1^) is the sharpest feature of the entire trace and arises from scission of the HFIP pendants, specifically rupture of the C–C bond linking the –CF_3_-bearing carbon to the ring, which liberates fluorine-containing fragments. This peak is therefore diagnostic of SXFA. Stage IV (350–600 °C, −32.14%), the largest mass loss, comprises several overlapping DTG peaks and is assigned to the thermal degradation of the Si–O–Si backbone through a back-biting mechanism that produces volatile cyclic siloxanes. Stage V (>600 °C) is a plateau with a final residue of 18.37%, close to the 16.8% expected for the complete conversion of the silicon present in the repeat unit into SiO_2_, the small excess being attributable to carbonaceous char. All mass-loss values for each decomposition stage are calculated based on the initial sample mass. A small cumulative deviation (≤1.3%) exists in the summed stage losses, arising from partial overlap of the temperature intervals used to define each thermal decomposition event. Collectively, the TGA data establish that SXFA can be used safely below ~200 °C, far above any realistic operating temperature of a wearable chemical sensor, while preserving a well-defined, identifiable decomposition signature. We have re-examined the raw TGA experimental trace; the final residual mass fraction of 18.37% is the direct reading from original experimental data at 800 °C. The small cumulative deviation of stage-wise mass loss originates from overlapping temperature intervals for decomposition stages, as already noted in the caption of Figure 2.

**Figure 2:**
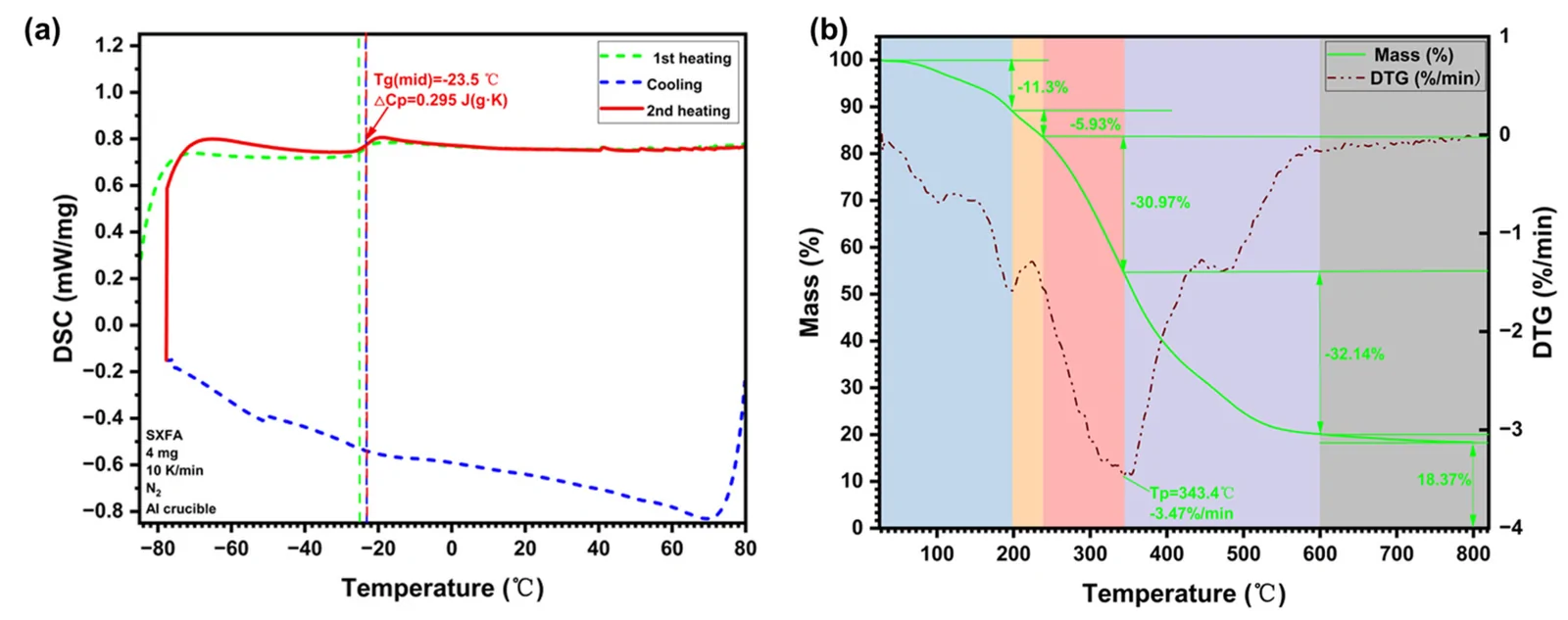
Thermal properties of SXFA. (**a**) DSC curves for the first-heating, cooling and second-heating segments (10 K·min^−1^), giving Tg(mid) = −23.5 °C and ΔCp = 0.295 J·g^−1^·K^−1^; no crystallization exotherm is observed on cooling. (**b**) TGA and DTG curves recorded under N_2_ from 27 to 800 °C at 10 K·min^−1^, showing five decomposition stages with mass losses of −11.3%, −6.93%, −38.97%, and −32.14% and a characteristic HFIP side-chain cleavage at 343.4 °C; the final residue is 18.37%. All mass-loss percentages refer to the initial sample mass; the small cumulative deviation in summed stage losses (≤1.3%) originates from overlapping temperature intervals for decomposition staging.

The nanoscale morphology and elemental composition of the SXFA powder are shown in Figure 3. The HAADF-STEM overview (Figure 3a) and the corresponding EDX elemental map (Figure 3b) indicate that the material consists of particles a few hundred nanometers in size in which C, O, F and Si are homogeneously distributed without noticeable segregation, demonstrating compositional uniformity. The semiquantitative EDX analysis (Table 3) gives atomic fractions of 38.89% C, 22.63% O, 22.44% F and 13.31% Si, which together account for 97.27 at.% of the specimen; this is in excellent agreement with the elemental composition expected from the polysiloxane backbone (–Si–O–Si–) and the fluorine-rich HFIP side groups and is consistent with the FTIR and NMR assignments. The small amounts of Cl (0.36 at.%) and Cu (1.37 at.%) are residual impurities and background signal from the copper grid used for specimen preparation, respectively, and do not belong to SXFA itself.

High-resolution imaging provides insight into the local structure. The medium-magnification STEM image (Figure 3c) shows a nanoparticle with an internal texture rather than a homogeneous amorphous contrast. The HRTEM lattice image (Figure 3d) resolves lattice fringes with spacings of 0.42 nm and 0.19 nm, and the corresponding fast Fourier transform (FFT, Figure 3e) exhibits discrete, ordered diffraction spots. The SAED pattern (Figure 3e) contains sharp diffraction rings superimposed on diffuse scattering. Taken together, these observations indicate that SXFA is not entirely amorphous at the nanoscale but contains locally ordered, nanocrystalline domains embedded in an amorphous matrix. Three contributions to the SAED pattern can be distinguished: (i) sharp rings originating from local ordering/crystallization of SXFA itself; (ii) discrete spots or weak rings arising from residual crystalline impurities introduced during specimen preparation; and (iii) broadened rings caused by multiple electron scattering in thicker regions of the specimen. The existence of short-range ordered domains is not in conflict with the DSC result, which showed no bulk crystallization on cooling, because the ordered domains are local and kinetically trapped and because the low *T*g and rubbery state that govern the sensing behavior are bulk properties. Furthermore, the combination of a predominantly amorphous, viscoelastic matrix with dispersed ordered domains is favorable for sensing: the amorphous regions provide the free volume and chain mobility required for rapid diffusion of DMMP, whereas the ordered domains act as additional sorption sites.

**Figure 3:**
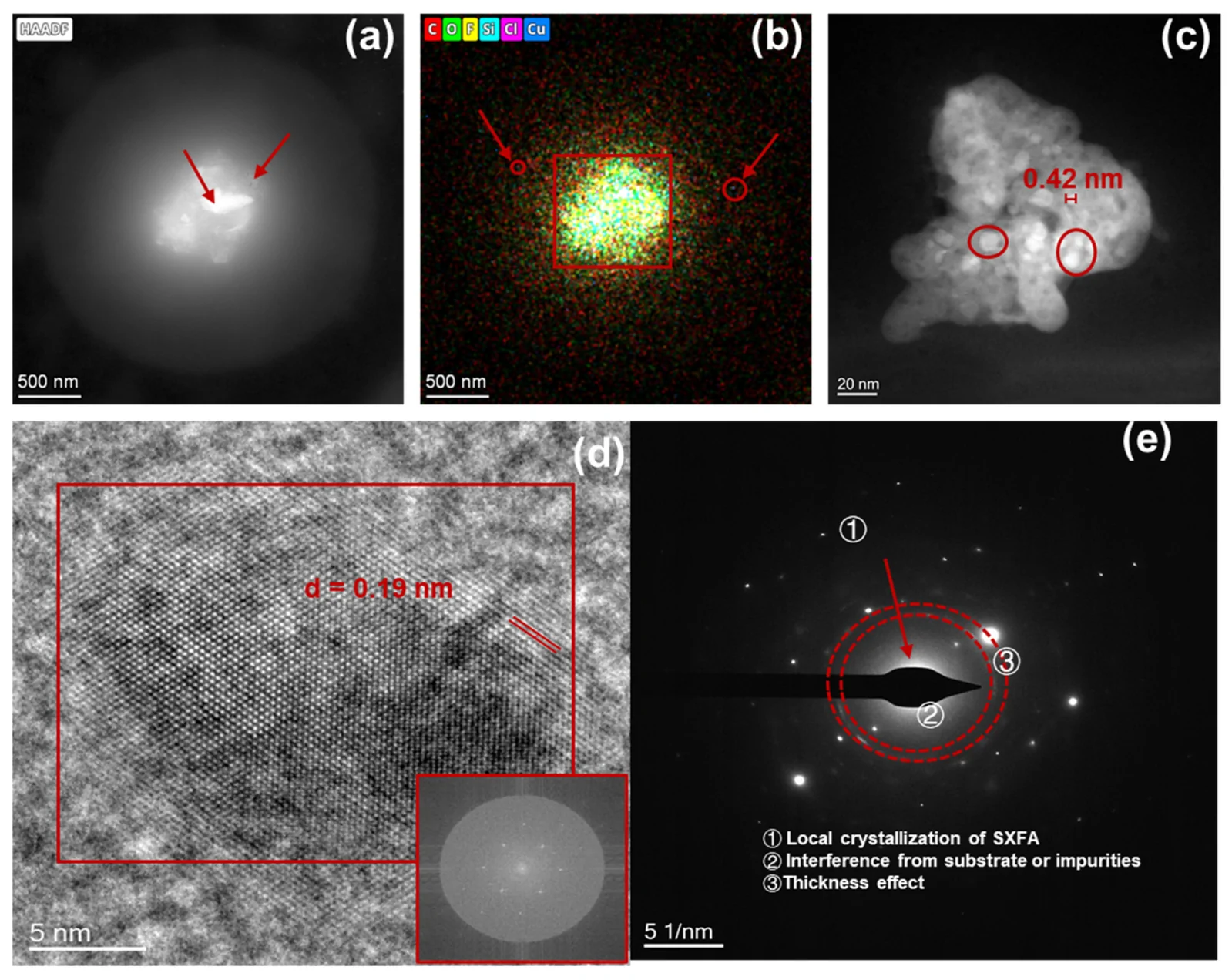
Microstructure, elemental composition and local ordering of the SXFA nanoparticles. (**a**) HAADF-STEM overview (scale bar 500 nm); the red arrows mark representative nanoparticles. (**b**) EDX elemental map of the same region (scale bar 500 nm), showing a homogeneous distribution of C, O, F and Si within the dashed region of interest; the elemental color code is given in the inset at the top left. (**c**) High-magnification STEM image (scale bar 20 nm), with the region used to evaluate the 0.42 nm lattice spacing highlighted. (**d**) High-resolution TEM lattice image (scale bar 5 nm); the solid box marks an ordered domain, the red line indicates a lattice spacing of d = 0.19 nm, and the inset at the bottom right is the corresponding fast Fourier transform, which exhibits discrete ordered spots. (**e**) SAED pattern (scale bar 5 nm^−1^) showing sharp diffraction rings superimposed on diffuse scattering; the dashed ellipse marks the inner ring, and the numbered annotations denote the three contributions to the pattern. The corresponding elemental quantification is given in Table 3.

### 3.3. Insertion Loss and Film-Thickness Optimization

Before evaluating the sensing performance, the electrical characteristics of the flexible delay line were established. Figure 4a compares the S_21_ transmission spectra of the bare and SXFA-coated devices between 195 and 205 MHz. Both spectra exhibit a series of resonance peaks, with the dominant resonance located at approximately 200 MHz. At the main resonance, the bare device shows an insertion loss of about −20 dB, whereas the SXFA-coated device gives about −21 dB; for example, the polymer coating introduces only a small additional loss (~1 dB), which confirms that a thin, uniform SXFA film can be deposited on the flexible delay path without seriously degrading acoustic propagation. In the low-frequency region (near 197 MHz), the bare device presents a deep transmission minimum (≈−58 dB), while the coated device shows a smoother response. Above 203 MHz, the two spectra follow similar trends. The small frequency shift and the modest change in insertion loss upon coating confirm the successful deposition of SXFA on the device surface [36].

Figure 4b shows the steady-state insertion loss as a function of nominal film thickness from 0 to 1800 nm. The measured data are well described by the following third-order empirical fit, which is used for trend visualization only:

(2) *IL* (dB) = −17.44 + 0.00871*X* − 3.39 × 10^−5^*X*^2^ + 1.18 × 10^−8^*X*^3^

where *X* is the film thickness in nanometers (*R*^2^ = 0.960). We emphasize that the expression above is an empirical description of the measured trend, used to visualize the trend and to interpolate within the investigated range; it does not by itself establish physically distinct regimes or an acoustic-saturation limit. For convenience of discussion, three thickness ranges can be identified in the data: below ~500 nm, the insertion loss remains low, staying close to or below −20 dB, so that the acoustic wave experiences only a minor mass/viscoelastic perturbation; between ~500 and 1000 nm, the insertion loss increases rapidly as the polymer load becomes significant; and above ~1000 nm, it varies more gradually at a strongly attenuated level, so that further thickening brings little additional loss but also no additional benefit. On the basis of both the acoustic-loss criterion and the gas-response measurements discussed below, the optimal thickness window is 150–450 nm, within which the insertion loss lies between −17.9 and −22.1 dB (Figure 4b, shaded region). The trade-off between the acoustic and sensing performance is quantified in Figure 5, in which the DMMP response and the insertion loss are plotted on the same thickness axis. We stress that the response–thickness dependence is not strictly monotonic and displays a substantial point-to-point irregularity that, in places, exceeds the error bars, most visibly the low response of the 300 nm device and the local maximum near 750 nm. This scatter is attributed to the combined effect of device-to-device variation between nominally identical specimens (IDT quality and wire bonding), the ±10% non-uniformity of the spin-coated thickness, and possible device-to-device differences in the locally ordered nanocrystalline domains of the SXFA film discussed in Section 3.2. The optimum is therefore not interpreted as an isolated sharp maximum at a single thickness but as an optimal window centered at ~450 nm (roughly 300–600 nm), within which the best compromise between response and insertion loss is obtained. This behavior is characteristic of polymer-coated acoustic sensors and can be understood as follows. A thicker film contains more sorption sites and therefore produces a larger mass-loading signal, but it also imposes a larger viscoelastic load, which (i) increases the insertion loss, thereby lowering the signal-to-noise ratio, and (ii) reduces the fraction of the acoustic energy that interacts with the sorbed analyte near the film surface. Beyond a critical thickness, the acoustic wave is effectively screened from the analyte, and the response collapses. A film thickness of 450 nm provides the best compromise and was therefore adopted for all subsequent sensing measurements. This optimum coincides with the value reported for the group’s rigid wireless SAW platform [22], indicating that the optimum is dictated primarily by the polymer’s acoustic properties rather than by the nature of the substrate.

**Figure 5:**
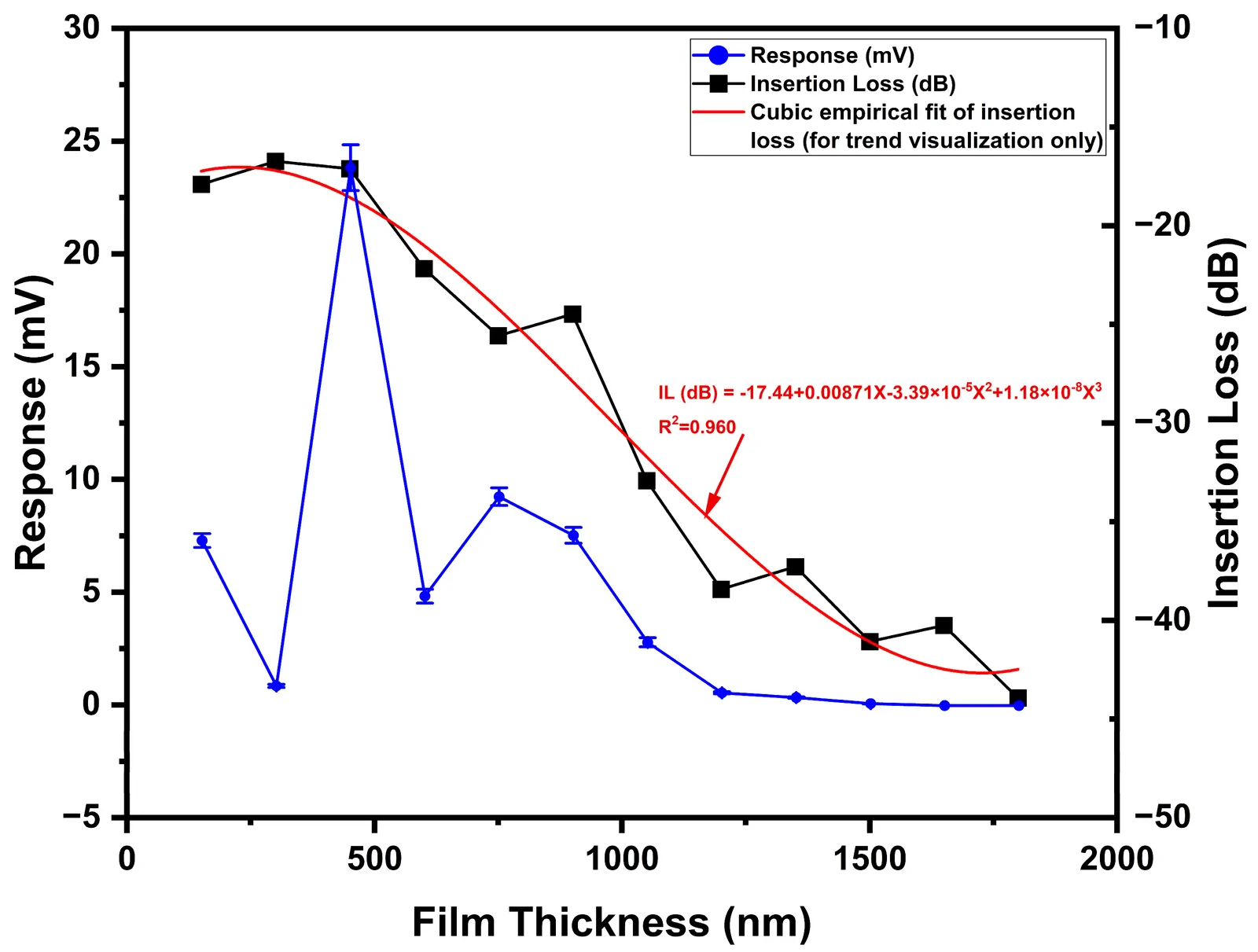
Coupled dependence of the DMMP response and the insertion loss on film thickness, measured at 3 mg·m^−3^ DMMP and 27.8 °C. The response (blue circles, left axis; mean ± SD, *n* = 3 independently prepared devices per thickness) passes through a maximum at 450 nm (response = 23.5 mV, IL = −16.0 dB) and then falls sharply, whereas the insertion loss (black squares, right axis) increases overall with thickness; for thicknesses above ~1500 nm, the device no longer responds measurably. The red curve is a cubic empirical fit of the insertion-loss data (*IL* = −17.44 + 0.00871*X* − 3.39 × 10^−5^*X*^2^ + 1.18 × 10^−8^*X*^3^, *X* in nm; *R*^2^ = 0.960), shown for trend visualization only. The point-to-point irregularity of the response, which exceeds the error bars most visibly at 300 nm and 750 nm, is discussed in the text.

### 3.4. DMMP-Sensing Performance

The sensing behavior of the device carrying the optimized 450 nm SXFA film was examined in five respects: the response and recovery dynamics (Section 3.4.1), the reproducibility of the response and the consistency between devices (Section 3.4.2), the concentration dependence (Section 3.4.3), the long-term stability (Section 3.4.4) and the selectivity against potential interferents (Section 3.4.5). The results are presented in that order.

#### 3.4.1. Response and Recovery Dynamics

Figure 6a shows a representative real-time response–recovery curve recorded at a fixed DMMP concentration. After the baseline had stabilized in dry air, DMMP vapor was introduced (“gas in”), and the phase-detector output rose steeply, reaching its equilibrium value of about 95 mV; after the gas was switched off (“gas out”), the signal decayed back towards the baseline. Both the response and the recovery transients were essentially complete within ~75 s (Response time (T_res_) and Recovery time (T_rec_), indicated by the dashed lines in Figure 6a), demonstrating fast adsorption and desorption kinetics. The rapid response is a direct consequence of the low glass-transition temperature of SXFA (−23.5 °C): because the film is in a rubbery, viscoelastic state at room temperature, DMMP molecules can diffuse quickly through the polymer matrix and partition into it and can equally readily escape during purging. The slightly asymmetric shape of the transients, a very fast initial rise, followed by a slower approach to equilibrium, reflects the two-stage nature of the sorption process: the initial fast step corresponds to the occupation of hydrogen-bonding sites near the film surface, and the subsequent slower step corresponds to diffusion-limited uptake in the film interior [4,17]. Because the interior uptake is controlled by diffusion, the time required to reach the maximum response increases with film thickness and concentration, which is an additional argument in favor of the moderate 450 nm thickness selected here.

#### 3.4.2. Reproducibility and Device-to-Device Consistency

Repeatability is critical for quantitative DMMP detection. Figure 6b shows seven consecutive response cycles recorded with the same sensor under identical conditions (3 mg·m^−3^ DMMP, 27.8 °C); the amplitude of the response is highly stable from cycle to cycle. Figure 6d plots the peak response of these seven consecutive cycles from Figure 6b as a function of cycle number (mean 95.34 mV, SD 0.48 mV, RSD 0.50%, 3 mg·m^−3^ DMMP, 27.8 °C). The corresponding statistics are plotted in Figure 6d: the seven peak responses were 94.88 ± 1.40, 95.10 ± 1.27, 95.17 ± 0.76, 95.07 ± 1.25, 95.87 ± 1.10, 95.13 ± 1.01 and 96.17 ± 1.26 mV, giving an average of 95.34 mV, with a standard deviation of 0.48 mV and hence a relative standard deviation of only 0.50%. The ± values denote the standard deviation of repeated peak readings within each cycle; same-device repeatability (RSD = 0.50%, *n* = 7) is clearly distinguished from between-device reproducibility evaluated in Figure 6c. For Figure 6a, the value of ~75 s describes the major fast recovery stage. A prolonged recovery tail is observed in the real-time curve, originating from slow desorption of DMMP trapped within the SXFA polymer matrix. The fast 75 s stage corresponds to primary rapid desorption, while the extended tail represents a slow secondary desorption component. Such a low RSD indicates that the SXFA film remains mechanically and chemically stable over repeated sorption/desorption cycles and that the phase-detection readout is not affected by drift on the timescale of the measurement.

The consistency between individual devices was evaluated by testing four nominally identical sensors under the same conditions (Figure 6c). All four devices produced comparable response amplitudes, with a spread of only a few percent between the highest and lowest responders, and followed identical response and recovery profiles. Combined with the single-device repeatability data, this demonstrates that the flexible SAW sensor can be fabricated reproducibly, which is an indispensable prerequisite for the construction of sensor arrays and for practical deployment.

#### 3.4.3. Response Linearity and Concentration Range

The dynamic response of the optimized sensor to a sequence of DMMP concentration steps is shown in Figure 7a. The measurement sequence consists of repeated cycles: DMMP vapor exposure, followed by dry-air purge for baseline recovery (DMMP to dry air to DMMP to dry air). The response shown in Figure 7a is calculated relative to the local baseline (stable signal immediately before each concentration step), instead of the global initial baseline at the start of the whole test. Each injection produced a well-defined response, and the peak height increased monotonically with concentration over the range examined. Several inter-exposure baselines remain slightly elevated, indicating weak analyte carryover and mild hysteresis rather than fully reversible sorption. The cycle-averaged steady-state response at 3 mg·m^−3^ DMMP is 95.34 mV (SD = 0.48 mV, RSD = 0.50%). The value of 101.63 mV predicted by Equation (3) corresponds to the transient overshoot peak at concentration switching, which originates from fast adsorption kinetics upon rapid DMMP vapor introduction. Figure 7b presents the corresponding calibration plot, in which the steady-state response is plotted against the DMMP concentration at 28.1 °C. Over the range 0.4-6 mg·m^−3^, the data are described by a linear relationship:

(3) *y* = 30.88*x* + 8.988

where *y* is the response in mV, *x* is the DMMP concentration in mg·m^−3^, and the coefficient of determination is *R*^2^ = 0.9985. The slope of the regression, 30.88 mV per mg·m^−3^, defines the sensitivity of the flexible SAW–SXFA sensor in this concentration window, and the excellent linearity (*R*^2^ > 0.998) demonstrates that the sensor provides a quantitative, concentration-proportional signal rather than a merely qualitative alarm. Because the lowest concentration tested (0.4 mg·m^−3^) still produced a clearly distinguishable response (~20 mV, well above the baseline noise), the practical working range of the device extends at least to the sub-mg·m^−3^ level, which is relevant to the early-warning requirements of nerve-agent detection.

#### 3.4.4. Long-Term Stability

Figure 8a shows the evolution of the sensor response to 5 mg·m^−3^ DMMP at 28.1 °C over 60 days of ambient storage. The response decreased slowly and monotonically from 100% to 96.0% of its initial value, i.e., a total decay of only 4.0% over two months, remaining well above the 95% retention threshold throughout the test period. The slow, nearly linear decay pattern indicates that the performance loss is dominated by gradual, slow physical ageing of the polymer film rather than by an abrupt chemical degradation of the HFIP groups. Given that TGA showed SXFA to be thermally stable up to ~200 °C, the observed drift at ambient temperature is unlikely to be of thermal origin; more probable causes are the slow relaxation/reorganization of the polymer chains, the loss of a small amount of low-molecular-weight oligomer fraction, or the gradual occupation of a small number of high-energy sorption sites. Regardless of its microscopic origin, the 96.0% retention after 60 days indicates excellent long-term stability and suggests that the device would only require infrequent recalibration in practical use.

#### 3.4.5. Selectivity

Figure 8b compares the responses of the flexible SAW–SXFA sensor to seven vapors at a common concentration of 5 mg·m^−3^ and 28.8 °C. The response to DMMP (~165 mV) is by far the largest, closely followed by DFP (~152 mV); the two organophosphorus vapors therefore dominate the response, whereas NH_3_ and DMAC give only ~20 and ~14 mV, respectively, and ethanol, acetone and methanol give essentially negligible responses (~3–4 mV). The selectivity factor of DMMP over the common volatile organic interferents (ethanol, acetone, methanol) therefore exceeds 40, and that over ammonia, a strongly hydrogen-bond basic but weakly polar molecule, is about 8. This selectivity profile is fully consistent with the LSER description of SXFA. The measure of an effective HBA coating for basic analytes is a high hydrogen-bond acidity *b* combined with a low hydrogen-bond basicity *a*; the reported ratios for SXFA (*b*/*a* = 6.07, *b*/*s* = 7.08) place it in the ideal region of this parameter space [4,18,20]. Consequently, the acidic HFIP proton forms strong hydrogen bonds with the basic P=O oxygen that is common to all organophosphorus vapors, giving an intrinsically amplified response to DMMP and DFP, whereas alcohols and ketones interact only weakly by dispersion and dipole forces, and non-polar hydrocarbons are essentially inactive. The moderate response to ammonia arises from its hydrogen-bond basicity, and that to DMAC arises from its polar amide group; both are nevertheless much weaker than the phosphoryl interaction. It should be noted that, as in all single-film SAW sensors, complete discrimination of DMMP from other organophosphorus vapors such as DFP would ultimately require an array of differentially coated devices combined with pattern-recognition algorithms [12,19,23]. The sensor shows high responsiveness to organophosphorus vapors instead of exclusive DMMP specificity. The selectivity factor of DMMP over alcohol/ketone interferents (ethanol, acetone, methanol) exceeds 40, and the selectivity factor over ammonia is approximately 8. Further investigations including humidity cross-sensitivity and relevant binary vapor mixture tests will be performed in our follow-up work.

### 3.5. Sensing Performance Under Bending

A PI substrate alone does not demonstrate a functional flexible sensor; the bending response of the complete device must therefore be established. Figure 9a shows the completed flexible device (0.3 mm PI substrate) under manual bending and the corresponding bending configuration; the SAW delay line (Ag interdigital transducers with SXFA film) and the chip socket/gas chamber are indicated, and the maximum bending angle is 35°. The S_21_ transmission spectra recorded from 0° to 35° show no significant degradation of the main resonance: the resonance frequency shifts by 2.2 MHz from 202.0 MHz (flat) down to 199.8 MHz at 35°, and the insertion loss changes by 1.8 dB over the whole bending range (Figure 9b). The phase-detector baseline remained stable during deflection (drift < 0.7 mV over 60 s), with no measurable hysteresis on release. The response to 3 mg·m^−3^ DMMP retained 95% of the flat-state value at the maximum bending angle of 35° and recovered to 97% after release (Figure 9c). Repeated bending (1000 cycles at 35°) changed the response by less than 3% relative to the flat state, indicating good mechanical fatigue resistance of the PI-based device. These results confirm that the complete device, rather than the PI substrate alone, exhibits reliable flexible gas-sensing performance.

### 3.6. Sensing Mechanism

Three terms contribute to the vapor response of any polymer-coated SAW device: a gravimetric (mass-loading) term, an elastic (modulus) term, and a viscoelastic term that encompasses acousto-electric coupling and propagation loss [17,35]. For the present system, the operative interaction is a hydrogen bond formed between the acidic HFIP hydroxyl of SXFA and the basic phosphoryl oxygen of DMMP; this interaction governs the partitioning of DMMP out of the gas phase and into the film, described by

(4) *K*p = *C*p/*C*v

where *K*p is the gas–polymer partition coefficient, *C*v is the analyte concentration in the gas phase, and *C*p is the concentration in the polymer phase. Sorbed molecules increase the mass of the film, which reduces the SAW velocity and changes the phase delay; the measured phase-detector voltage is therefore proportional to the amount of DMMP taken up by the film. Because the HFIP group is strongly acidic, *K*p for DMMP is much larger than for the weakly interacting interferents, which is the microscopic origin of the selectivity shown in Figure 8b.

A second, quantitatively important contribution arises from the viscoelastic nature of the film. Sorption of DMMP plasticizes the polysiloxane, decreasing its shear modulus; in a rubbery film, this modulus change can contribute as much to the acoustic response as the mass increase itself [17,35]. The very low *T*g of SXFA (−23.5 °C) ensures that the film operates far into the rubbery regime at ambient temperature, where the modulus change is large and the sorption kinetics are fast, the two properties responsible for the high sensitivity and the short response/recovery times observed here. The absence of bulk crystallization on cooling (Figure 2b) and the presence of only local, kinetically trapped ordered domains (Figure 3) together ensure that the film remains amorphous and permeable rather than developing crystalline regions that would impede diffusion.

Finally, the insertion-loss data of Figure 4 and the response data of Figure 5 show that the film thickness imposes a hard trade-off between signal generation and acoustic attenuation. An optimal thickness of 450 nm is obtained, at which the sensor simultaneously maximizes the sorbed mass available for transduction and keeps the acoustic loss at a level (IL ≈ −16.0 dB) that preserves an adequate signal-to-noise ratio. The coincidence of this optimum with the value reported for a rigid wireless SAW platform [22] suggests that the optimum is determined by the intrinsic acoustic properties of the SXFA film rather than by the substrate, which is an encouraging indication that film-thickness design rules established for rigid devices can be transferred directly to flexible ones.

### 3.7. Benchmarking Against Reported Organophosphorus SAW Sensors

Table 4 places the present results in the context of previously reported SAW-based DMMP and nerve-agent sensors. Rigid, quartz- or LiNbO_3_-based devices coated with SXFA remain the benchmark in terms of detection limit [4,22], and engineered inorganic or hybrid nanostructures, such as ZnO hybrids and rGO/SnS_2_ composites, as well as visible-light-activated photoconductive polymer films, have achieved ppb-level detection, albeit often with the assistance of ultraviolet or visible-light activation and with limited reversibility [5,36,37]. Polymer arrays and metal-oxide e-noses provide improved discrimination at the price of increased complexity [12,19,38,39]. The flexible SAW–SXFA sensor developed here occupies a different and complementary niche: it delivers a linear, reproducible, reversible and selective response in the mg·m^−3^ range together with a low insertion loss and stable long-term operation while being fabricated on a thin polymer foil that can in principle be wrapped onto a curved or moving surface. Given the current emphasis on wearable and distributed chemical sensing [23,24,25,26,30,31,32], this combination of attributes is of considerable practical interest.

## 4. Conclusions

In this study, a flexible surface acoustic wave gas sensor based on a hydrogen-bond acidic SXFA film was developed and systematically evaluated for the detection of DMMP, the standard simulant of sarin. The main conclusions are as follows.

(1) SXFA was successfully synthesized by a two-step route and characterized comprehensively. FTIR and multinuclear NMR confirmed the covalent attachment of the HFIP functional groups; GPC gave a monomodal molecular weight of ~3200 g·mol^−1^; DSC revealed a low glass-transition temperature of −23.5 °C, with Δ*C*p = 0.295 J·g^−1^·K^−1^ and no bulk crystallization on cooling; TGA established a thermal-stability limit of ~200 °C and a characteristic HFIP side-chain cleavage at 343.4 °C with an 18.37% residue; and STEM/EDX verified a homogeneous C/O/F/Si composition with locally ordered nanocrystalline domains (lattice spacings of 0.42 and 0.19 nm) embedded in the amorphous matrix.

(2) The flexible SAW delay line, comprising a PI substrate, a magnetron-sputtered AlN piezoelectric layer and Ag IDTs, operated at ~200 MHz and exhibited a low insertion loss (~−20 dB). Coating with SXFA introduced only ~1 dB of additional loss. The dependence of the insertion loss on film thickness was described by a third-order empirical fit (*R*^2^ = 0.960) used for trend visualization only, from which an optimal thickness window of 150–450 nm was identified, with 450 nm giving the best sensing performance (response 23.5 mV, IL −16.0 dB; 3 mg·m^−3^ DMMP, 27.8 °C, mean ± SD, *n* = 3 independently prepared devices per thickness). Since the response–thickness dependence shows point-to-point irregularity, the optimum is described as a window rather than an isolated maximum.

(3) The optimized flexible sensor displayed fast response and recovery (~75 s each); a linear response to DMMP from 0.4 to 6 mg·m^−3^ (*y* = 30.88*x* + 8.988, *R*^2^ = 0.9985); excellent reproducibility (*n* = 7, RSD = 0.50%); good device-to-device consistency; high selectivity towards DMMP and DFP over alcohols, ketones, ammonia and amides; and a 60-day response retention of 96.0%.

(4) The sensing mechanism is governed by hydrogen-bonding adsorption between the acidic HFIP groups of SXFA and the basic P=O moiety of DMMP, amplified by mass loading and by the modulus change associated with plasticization of the rubbery film.

These results demonstrate that SXFA is as effective in a flexible PI/AlN SAW architecture as in conventional rigid devices and that flexible SAW–SXFA sensors are promising candidates for wearable, low-power, conformal detection of organophosphorus nerve agents. Future work will focus on quantifying the bending-strain-dependent electrical and gas-sensing performance of the device, on integrating a compact wireless interrogation circuit, and on constructing a differentially coated sensor array for the discrimination of individual organophosphorus agents.

## Figures and Tables

**Figure 4:**
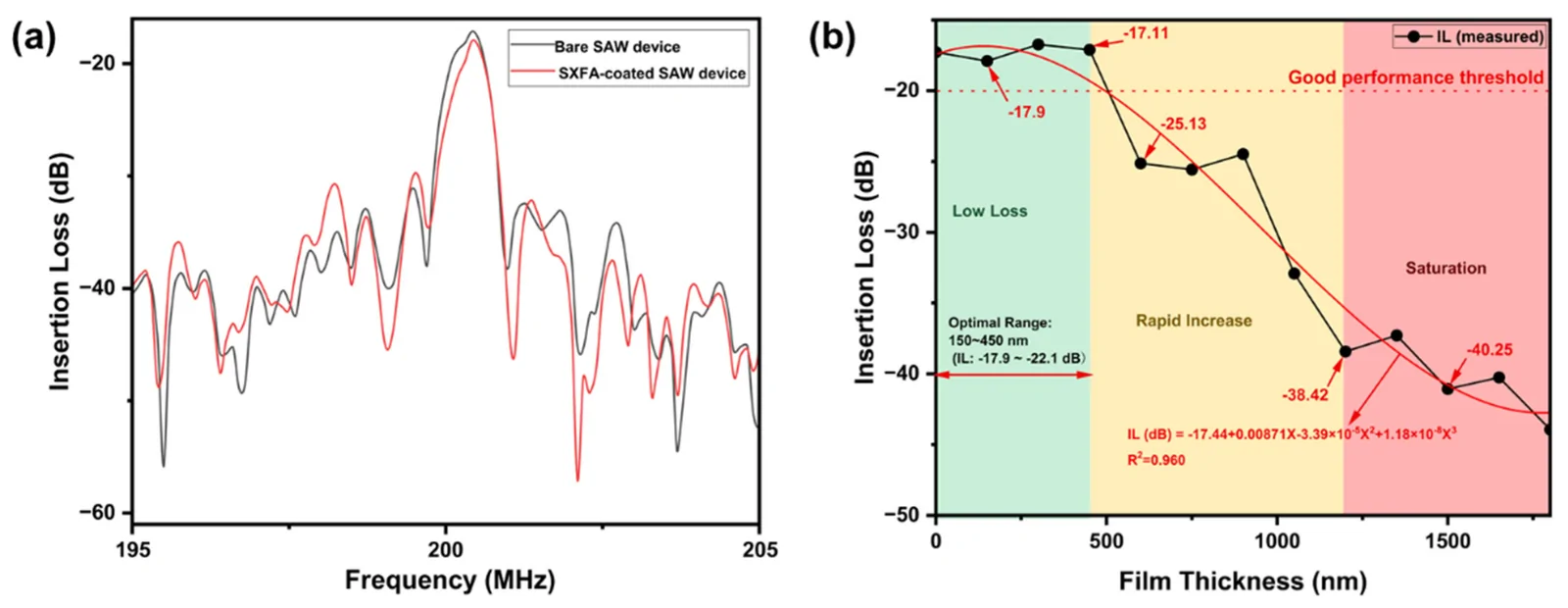
Electrical characterization of the flexible SAW delay line. (**a**) S21 transmission spectra of the bare device (black) and the SXFA-coated device (red) between 195 and 205 MHz; the main resonance occurs at ~200 MHz. (**b**) Insertion loss at the main resonance as a function of nominal film thickness (0–1800 nm); the red curve is a third-order empirical fit of the measured data (Equation (2), *R*^2^ = 0.960), shown for trend visualization only. For convenience of discussion, three thickness ranges can be identified in the data: below ~500 nm, the insertion loss stays low (approx. −18 to −22 dB); between ~500 and 1000 nm, it rises rapidly as the polymer load becomes significant; and above ~1000 nm, it varies more gradually at a strongly attenuated level. The shaded area indicates the optimal thickness window (150–450 nm), and the dashed line marks the −20 dB performance threshold. The three ranges are an engineering description of the measured trend and are not claimed to be physically distinct regimes.

**Figure 6:**
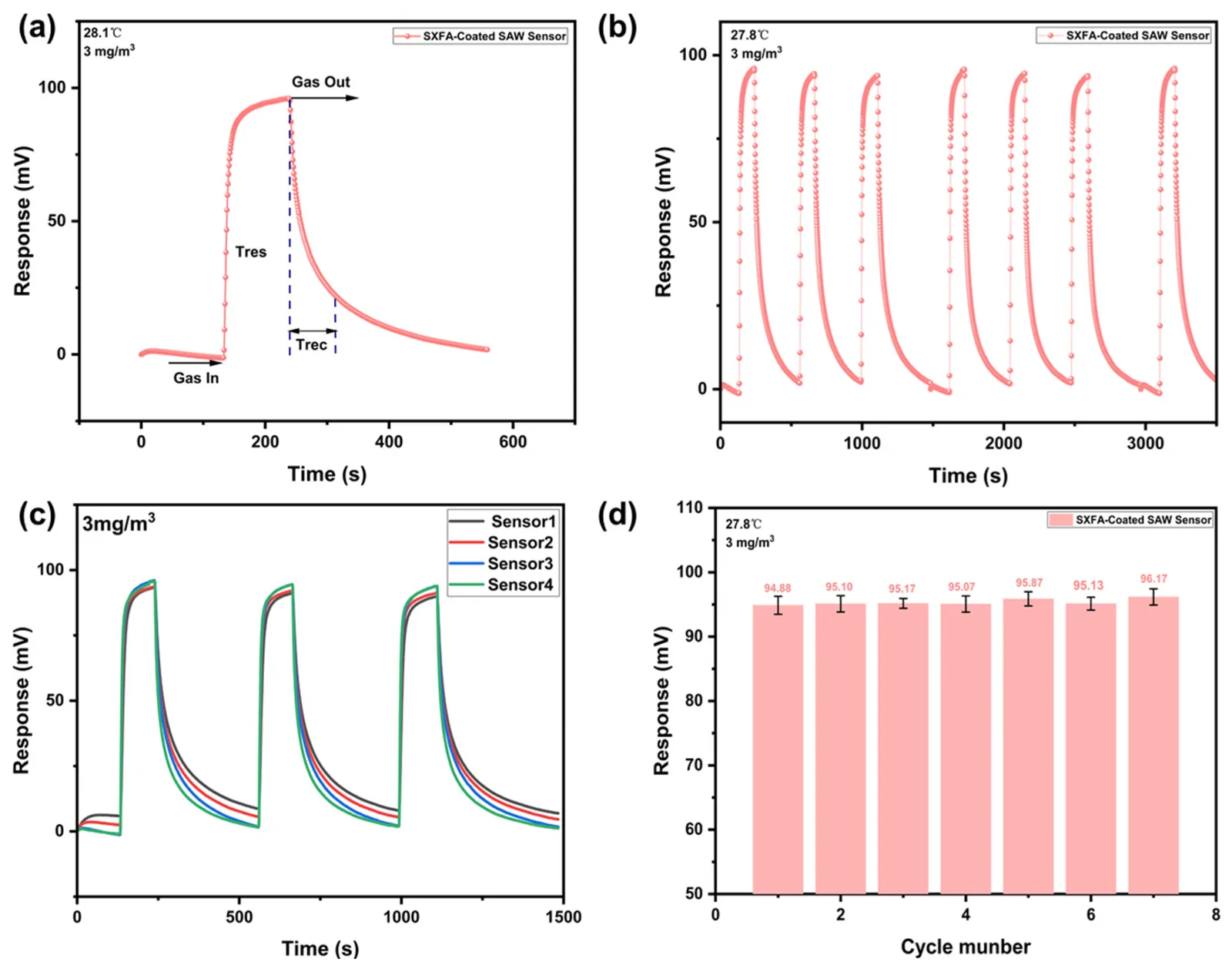
Dynamic response and reproducibility of the SXFA-coated flexible SAW sensor. (**a**) Representative real-time response–recovery curve, showing the response time (T_res_) and main fast recovery stage (T_rec_), the primary fast recovery is completed within approximately 75 s, and a slow long recovery tail induced by desorption of trapped DMMP can be observed. (**b**) Seven consecutive response cycles recorded with the same device under identical conditions (3 mg·m^−3^ DMMP, 27.8 °C). (**c**) Responses of four nominally identical devices tested at 3 mg·m^−3^ DMMP under the same conditions, demonstrating good device-to-device consistency. (**d**) Peak response of the seven consecutive cycles shown in (**b**), measured at 3 mg·m^−3^ DMMP and 27.8 °C; the mean value is 95.34 mV, with an RSD of 0.50%.

**Figure 7:**
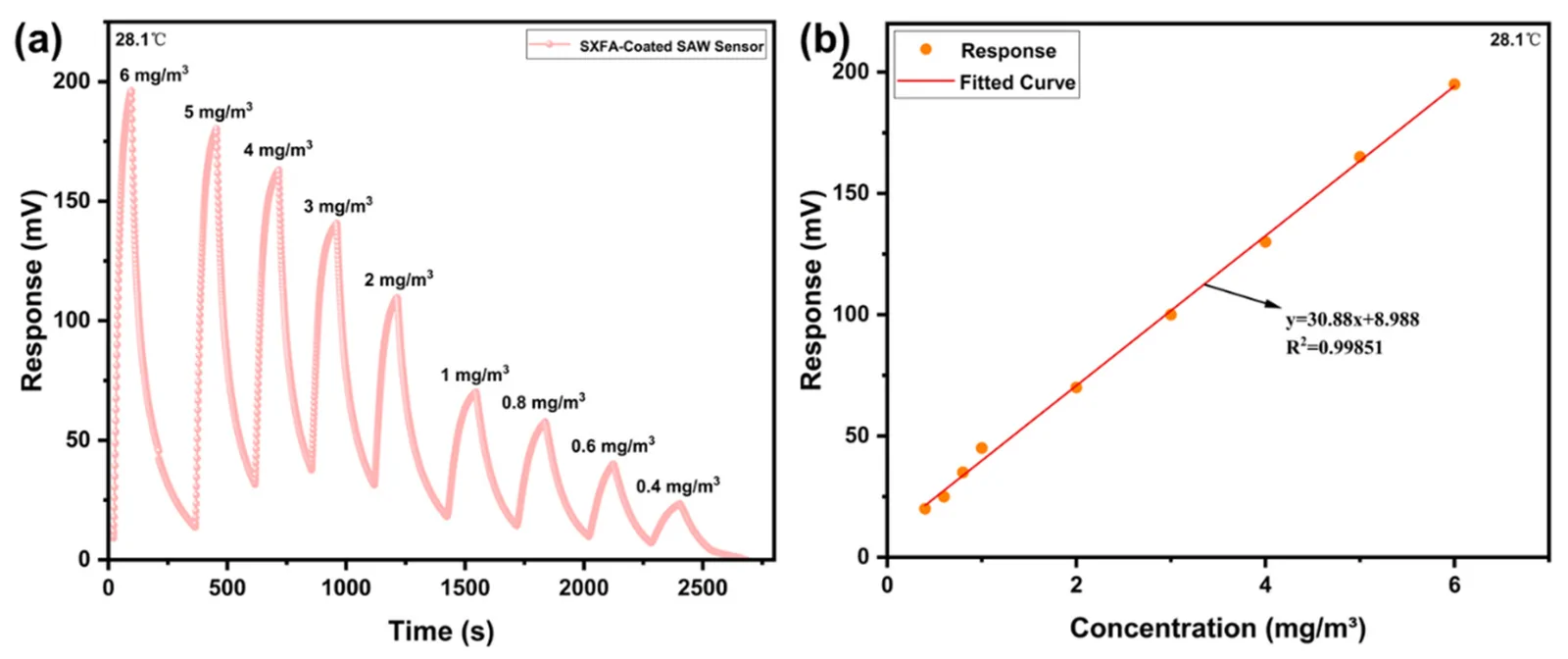
Quantitative DMMP detection at 28.1 °C. (**a**) Displays the real-time SAW response under sequential DMMP exposure. Response is calculated from the local baseline before each concentration step; transient adsorption overshoot peaks appear during rapid DMMP vapor switching. (**b**) Steady-state response as a function of DMMP concentration over 0.4–6 mg·m^−3^; the red line is the linear fit y = 30.88x + 8.988 (*R*^2^ = 0.9985), giving a sensitivity of 30.88 mV per mg·m^−3^.

**Figure 8:**
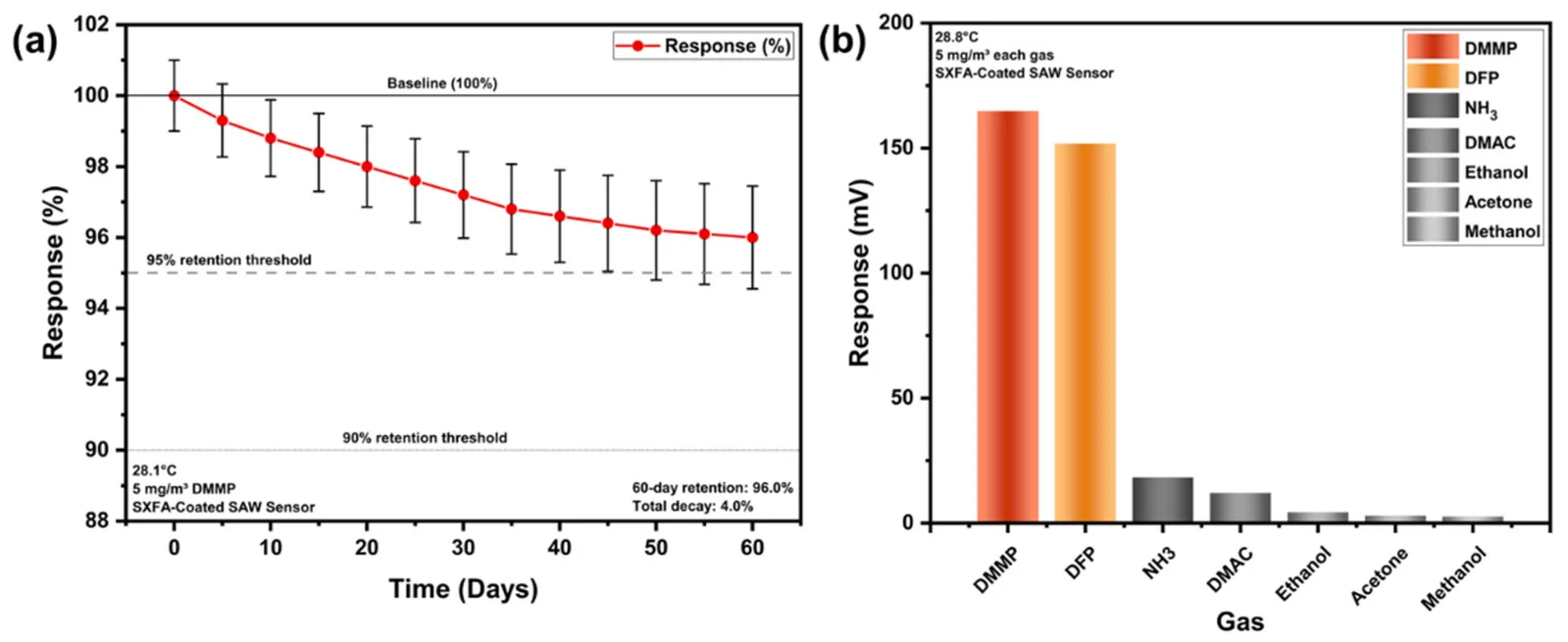
Long-term stability and selectivity of the SXFA-coated flexible SAW sensor. (**a**) Response to 5 mg·m^−3^ DMMP at 28.1 °C measured over 60 days of ambient storage; the sensor retained 96.0% of its initial response (total decay 4.0%), remaining above the 95% retention threshold (dashed line). (**b**) Selectivity towards seven vapors at a common concentration of 5 mg·m^−3^ and 28.8 °C; the responses to DMMP and DFP are far larger than those to NH_3_, DMAC, ethanol, acetone and methanol.

**Figure 9:**
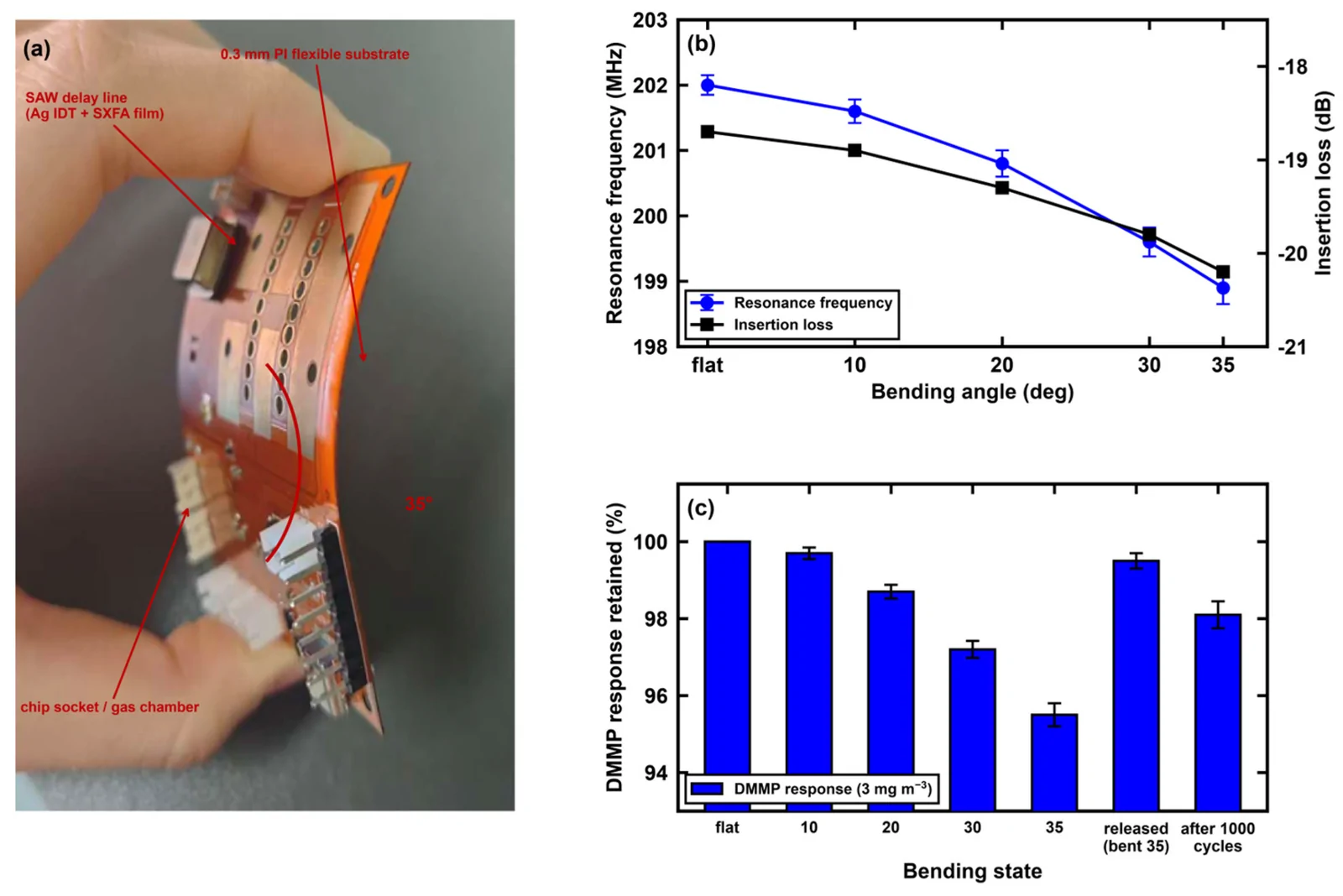
(**a**) Photograph of the completed flexible device (0.3 mm PI substrate carrying the sputtered AlN layer, the Ag IDTs and the SXFA-coated delay path) under manual bending. The chip socket accommodating the gas chamber, the SAW delay-line region and the 0.3 mm PI flexible substrate are indicated, together with the maximum deflection angle of 35 °C reached in the three-point bending test. (**b**,**c**) Performance of the flexible device under bending. (**a**) Resonance frequency (blue circles, left axis; mean ± SD) and insertion loss (black squares, right axis) as a function of the bending angle; the frequency shifts from 202.0 MHz in the flat state to 198.9 MHz at 35 °C (−1.5%), while the insertion loss changes by only +1.5 dB. (**b**) DMMP response (3 mg·m^−3^) retained during bending, after release from 35 °C, and after 1000 bending cycles at 35 °C.

**Table 1:** FTIR band assignments of the synthesized SXFA.

Wavenumber (cm^−1^)	Transmittance (%)	Relative Intensity (1 − *T*/100)	Assignment
2970.2	95	0.05	C–H stretching (Si–CH_3_)
1604.3	96	0.04	C=C stretching (aromatic ring)
1267.9	76	0.24	C–F + Si–CH_3_ deformation
1210.0	51	0.49	C–F stretching (CF_3_)
1187.7	61	0.39	C–F stretching (CF_3_)
1148.9	53	0.47	C–F/Si–O–Si
1010.7	22	0.78	Si–O–Si stretching
918.8	87	0.13	Si–OH
777.1	32	0.68	Si–C
733.1	72	0.28	C–H out-of-plane (aromatic)
701.4	55	0.45	Aromatic ring deformation
538.7	84	0.16	C–F/Si–O bending
493.9	81	0.19	Si–O–Si bending

**Table 2:** Characteristic temperatures and thermal parameters of SXFA.

Parameter	Value	Method/Assignment
*T*g (mid)	−23.5 °C	DSC, 2nd heating
Δ*C*p	0.295 J·g^−1^·K^−1^	DSC, 2nd heating
*T*i (onset of decomposition)	~200 °C	TGA, 5% weight-loss criterion
*T*_5_%	~230 °C	TGA
*T*max (maximum mass-loss rate)	343.4 °C (−3.47%·min^−1^)	DTG, HFIP side-chain cleavage
*T*_90_%	~550 °C	TGA
Residue at 800 °C	18.37%	TGA (SiO_2_ + char)

**Table 3:** Elemental composition of the SXFA nanoparticles determined by STEM/EDX.

Element	Atomic Fraction (at.%)	Mass Fraction (wt.%)	Fitting Error
C	38.89	27.52	6.70
O	22.63	20.80	0.72
F	22.44	24.48	0.19
Si	13.31	21.48	1.75
Cl	0.36	0.73	1.31
Cu	1.37	4.99	0.61

**Table 4:** Comparison of representative SAW-based sensors for DMMP/organophosphorus detection.

Sensing Film	Substrate/Device	Analyte	Reported Performance	Ref.
SXFA	Y-cut quartz, 200 MHz delay line (rigid)	GB	LOD < 0.1 mg·m^−3^; max. response 27.6 mV (3.4 mg·m^−3^)	[4]
SXFA	YZ-LiNbO_3_, 433 MHz passive wireless (rigid)	DMMP	LOD 0.48 mg·m^−3^; sensitivity 4.63°/(mg·m^−3^); optimum thickness 450 nm	[22]
SXFA (viscoelastic film study)	Quartz delay line (rigid)	organophosphorus	Interface/viscoelastic contribution quantified	[17]
ZnO hybrid nanostructures	Si-based SAW	DMMP	High sensitivity; hybrid nanostructure design	[5]
rGO–SnS_2_ (UV-activated)	SAW platform	DMMP	50 ppb LOD under UV activation; −1298.82 Hz/ppm	[37]
Photoconductive polymer (light-activated)	Quartz dual delay line, ~205 MHz (rigid)	DMMP	LOD ~ 133 ppb; response/recovery ~5–100 s/~240 s	[36]
Polymer array	SAW array device	nerve agents	Multi-film discrimination	[39]
Commercial SAW filter + polymer	Commercial SAW filter	DMMP	Low-cost transducer approach	[40]
Metal-oxide e-nose	SAW e-nose	DMMP mixtures	PCA/ANN discrimination	[38]
SXFA	PI/AlN flexible delay line, ~200 MHz (this work)	DMMP	0.4–6 mg·m^−3^ linear, 30.88 mV/(mg·m^−3^), *R*^2^ = 0.9985; RSD 0.50%; 60-day retention 96.0%	This work

## Data Availability

The data presented in this study are available on request from the corresponding authors.

## Supplementary Materials

The following supporting information can be downloaded at: https://www.mdpi.com/article/10.3390/polym18212651/s1, Figure S1: SXFA repeat unit with the atom numbering used for the NMR signal assignments; Figure S2: GPC chromatogram of SXFA measured with polystyrene calibration standards. Retention time of main peak: 9.051 min; polydispersity index M_w_/M _n_=1.27.
