# Supplementary material for: Flexible Surface Acoustic Wave Sensor Based on an SXFA Sensitive Film for DMMP Detection
Source: Polymers (Basel). 2026 Oct 26;18(21):2651. doi: 10.3390/polym18212651 (PMC13766186; doi:10.3390/polym18212651)
Supplement: Supplementary file 1 [file polymers-18-02651-s001.zip › polymers-4638714-supplementary.pdf]

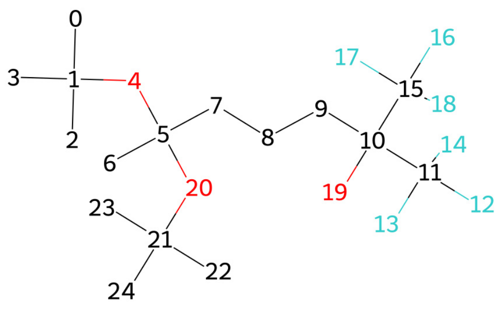

Figure S1. SXFA repeat unit with the atom numbering used for the NMR signal assignments.

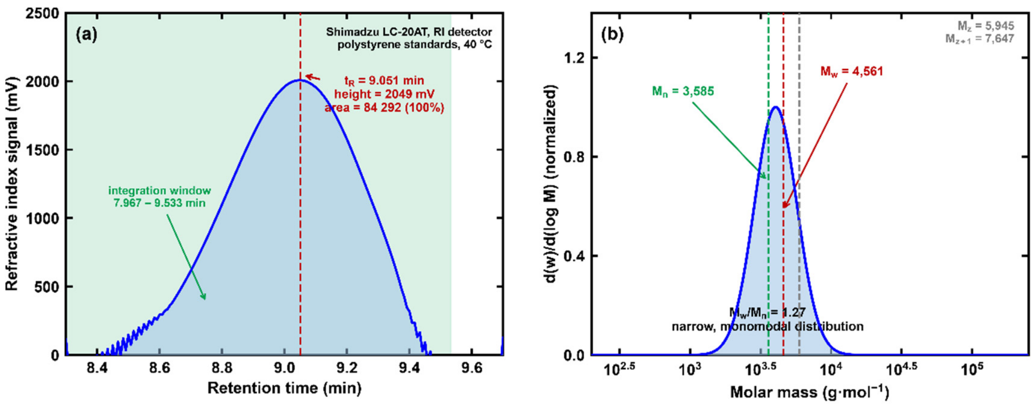

Figure S2. GPC chromatogram of SXFA measured with polystyrene calibration standards. Retention time of main peak: 9.051 min; polydispersity index M_w/M_n=1.27.
